# Utilizing Infrared Spectroscopy to Analyze the Interfacial Structures of Ionic Liquids/Al_2_O_3_ and Ionic Liquids/Mica Mixtures under High Pressures

**DOI:** 10.3390/nano9030373

**Published:** 2019-03-05

**Authors:** Yen-Hsu Chang, Hai-Chou Chang, Yen-Pei Fu

**Affiliations:** 1 Department of Chemistry, National Dong Hwa University, Shoufeng, Hualien 974, Taiwan; 410412017@gms.ndhu.edu.tw; 2 Department of Materials Science and Engineering, National Dong Hwa University, Shoufeng, Hualien 974, Taiwan; ypfu@gms.ndhu.edu.tw

**Keywords:** ionic liquids, infrared, spectroscopy, high pressures

## Abstract

The interfacial interactions between ionic liquids (1,3-dimethylimidazolium methyl sulfate and 1-ethyl-3-methylimidazolium trifluoromethanesulfonate) and solid surfaces (mesoporous aluminum oxide and mica) have been studied by infrared spectroscopy at high pressures (up to 2.5 GPa). Under ambient pressure, the spectroscopic features of pure ionic liquids and mixtures of ionic liquids/solid particles (Al_2_O_3_ and mica) are similar. As the pressure is increased, the cooperative effect in the local structure of pure 1,3-dimethylimidazolium methyl sulfate becomes significantly enhanced as the imidazolium C–H absorptions of the ionic liquid are red-shifted. However, this pressure-enhanced effect is reduced by adding the solid particles (Al_2_O_3_ and mica) to 1,3-dimethylimidazolium methyl sulfate. Although high-pressure IR can detect the interactions between 1,3-dimethylimidazolium methyl sulfate and particle surfaces, the difference in the interfacial interactions in the mixtures of Al_2_O_3_ and mica is not clear. By changing the type of ionic liquid to 1-ethyl-3-methylimidazolium trifluoromethanesulfonate, the interfacial interactions become more sensitive to the type of solid surfaces. The mica particles in the mixture perturb the local structure of 1-ethyl-3-methylimidazolium trifluoromethanesulfonate under high pressures, forcing 1-ethyl-3-methylimidazolium trifluoromethanesulfonate to form into an isolated structure. For Al_2_O_3_, 1-ethyl-3-methylimidazolium trifluoromethanesulfonate tends to form an associated structure under high pressures.

## 1. Introduction

Aluminum oxide, also known as alumina, is utilized in various applications. For instance, it can be used as the major component in ceramics, catalysis, paints, lubricants, and medical products [1,2,3,4,5,6,7,8]. Moreover, nanoporous anodic alumina can be obtained from electrochemical anodization of aluminum foil. This material has highly ordered and monodisperse pores, a large surface area, and many more useful properties [9]. Therefore, porous alumina can be used as filters with high selectivity for sensing ions or biomolecules [10,11,12,13,14]. Additionally, porous alumina can be chemically functionalized with 3-(mercaptopropyl)-trimethoxysilane (MPTMS), for example, for further modification to enable it to detect the amount of mercury ions in water [14]. Similarly, the adsorption of ionic liquids on porous alumina has the potential to improve the performance of these materials in industry. However, even though porous alumina has many applications, the interactions and physical arrangement of ionic liquids at porous alumina surfaces are still poorly understood, although some studies have shown that certain ionic liquid and porous alumina mixtures have complicated interfacial interactions [15].

Mica is a generic term for potassium aluminosilicates, with a number of isomorphic substitutions. The chemical structures of micas contain aluminum, which substitutes some silicon atoms. As a result, this leads to a high quantity of negative charge at mica surfaces [16]. A lot of research has indicated that ionic liquids can form layered structures at mica surfaces. These ionic liquid nanostructures near the solid surfaces may be induced by surface charge interactions [16,17,18,19]. Furthermore, there is experimental evidence that reveals that both the charge density and total density of ionic liquids vary periodically, or oscillate, with distance from the mica surface. This also can be observed on sapphire surfaces. Yet, for graphene surfaces (neutral materials), a mixed cation/anion layering with an interfacial densification of the first ionic liquid layer is revealed by combined high resolution X-ray interface scattering and MD simulation [20]. Thus, the layered structures of ionic liquids are different at solid surfaces with and without electrostatic interactions. Understanding the local structures of ionic liquids at surfaces is crucial to probe and control the interactions of ionic liquids at electrode surfaces in energy storage devices. For a fundamental understanding of the effect of charged surface on the ionic multilayer structures of ionic liquids, various vibrational spectroscopic techniques have been applied such as infrared reflection absorption spectroscopy [21], sum-frequency generation spectroscopy [22], surface-enhanced Raman scattering spectroscopy [23], and surface-enhanced infrared absorption spectroscopy [24]. Spectroscopic studies elucidate the mechanism of the hysteretic anion-cation exchange in the first ionic layer on electrodes [25,26]. For example, large-sized ions of ionic liquids may lead to higher activation barriers for the ion replacement in the first layer (the ion size effect) [27]. Actually, the hysteretic behavior of the interfaces has only been reported in spectroscopic studies [25]. Since the hysteretic behavior of the interfaces has been studied in recent years, deep insight into the effect of the hysteretic behavior on different surfaces such as charged/uncharged surfaces is still needed. In addition, hysteretic behavior makes it difficult to understand what happen if charged/uncharged surfaces are faced to ionic liquids. Therefore, it is important to investigate the differences in the properties of ionic liquids at mica and porous alumina interfaces in this study.

Ionic liquids are salts with negligible vapor pressure, and this property prevents them from polluting the environment by evaporation. In addition, they have intrinsically high ionic conductivity and low flammability. Hence, these fluids can be used as electrolytes in energy devices [28,29]. Furthermore, these characteristics give them the potential to serve as safer alternatives to traditional volatile organic solvents [30]. Additionally, the physicochemical properties of ionic liquids can be tuned by changing the combination of cations and anions. For instance, introducing bulky asymmetric cations, such as imidazolium or phosphonium, into ionic liquids can prevent them from packing through mismatch. Consequently, ionic liquids of this type usually have melting points lower than ambient temperature—the so-called room-temperature ionic liquids (RTILs) [31,32]. Many experiments have been conducted recently to probe the structures of these liquids at solid surfaces. Some results have indicated that the fluid structures at the interfaces are possibly controlled by interactions including hydrogen bonding, electrostatic forces, and van der Waals forces. Another possible factor that can influence these structures is the steric effect [33,34,35]. A lot of research has focused on changing the lengths of the alkyl chains on the cations to probe the arrangements of ionic liquids at solid surfaces. However, the effect that the ionic liquids’ anion type contributes to the structure is still unclear.

Many reports have revealed that the frequencies and signal intensities of the vibration modes of cations and anions in ionic liquids are sensitive to their potential energy environments [28,29,35]. In addition, the changes of these frequencies and intensities can be observed by infrared (IR) spectroscopy. Therefore, IR methodologies may be useful to probe the structures of ionic liquids at solid surfaces. Furthermore, it can be seen from some studies that there are weak hydrogen bonds between the aromatic C–H in the cations and the anions [36,37,38,39,40]. Moreover, the contribution to charge-assisted hydrogen bond energy by coulombic interactions is especially significant and the C–H…O hydrogen bonding in 1,3-dimethylimidazolium methyl sulfate is one of the dominating interactions [32]. Similarly, the strength of the hydrogen bond can also be studied by using infrared spectroscopy.

By changing the pressure applied to mixtures of ionic liquids and solids, the intramolecular forces at the liquid/solid interfaces can be tuned and interferences that are produced by changes in temperature and chemical composition at the same time can be avoided [41]. Consequently, the local structures of the fluids may be elucidated by observing the frequency shifts and changes in signal intensities of the ions’ vibrational signals under different pressure conditions.

## 2. Materials and Methods

Samples were prepared by using 1,3-dimethylimidazolium methyl sulfate (>97%, Fluka, Morris Plains, NJ, USA), 1-ethyl-3-methylimidazolium trifluoromethanesulfonate (99%, UniRegion Bio-Tech, Taoyuan, Taiwan), mica (a gift from Pei Long Enterprise, Taiwan), and mesoporous aluminum oxide (porous Al_2_O_3_, pore size 58 Å, 150 mesh, Sigma-Aldrich, St. Louis, MO, USA). The solid powders (~0.005 g) were mixed with ionic liquids (~0.1 g), followed by centrifugation, washing of the precipitate with ethanol (1 mL), centrifugation, and drying with a moisture analyzer (MS-70, A&D Company, Tokyo, Japan). A diamond anvil cell (DAC) of Merrill-Bassett design with a diamond culet size of 0.6 mm was used to generate pressures of up to ~2 GPa. Two type IIa diamonds were used for mid-infrared measurements. Infrared spectra of the samples were measured with a Fourier transform spectrophotometer (Spectrum RXI, Perkin-Elmer, Naperville, IL, USA) equipped with an LITA (lithium tantalite) mid-infrared detector. The infrared beam was condensed through a 5× beam condenser onto the sample in the diamond anvil cell. To remove the absorption of the diamond anvils, the absorption spectra of the DAC were measured first and subtracted from those of the samples. Samples were contained in a 0.3-mm-diameter hole in a 0.25-mm-thick Inconel gasket mounted on the DAC. To reduce the absorbance of the samples, CaF_2_ crystals were placed into the holes and compressed to be transparent prior to inserting the samples. The sample filled the entire empty space of the gasket hole in the DAC, which was subsequently sealed when the opposing anvils were pushed toward one another. Typically, we chose a resolution of 4 cm^−1^ (data point resolution of 2 cm^−1^). For each spectrum, 1000 scans were compiled. Pressure calibration was performed by following Wong’s method [42,43].

## 3. Results and Discussion

**Figure 1:**
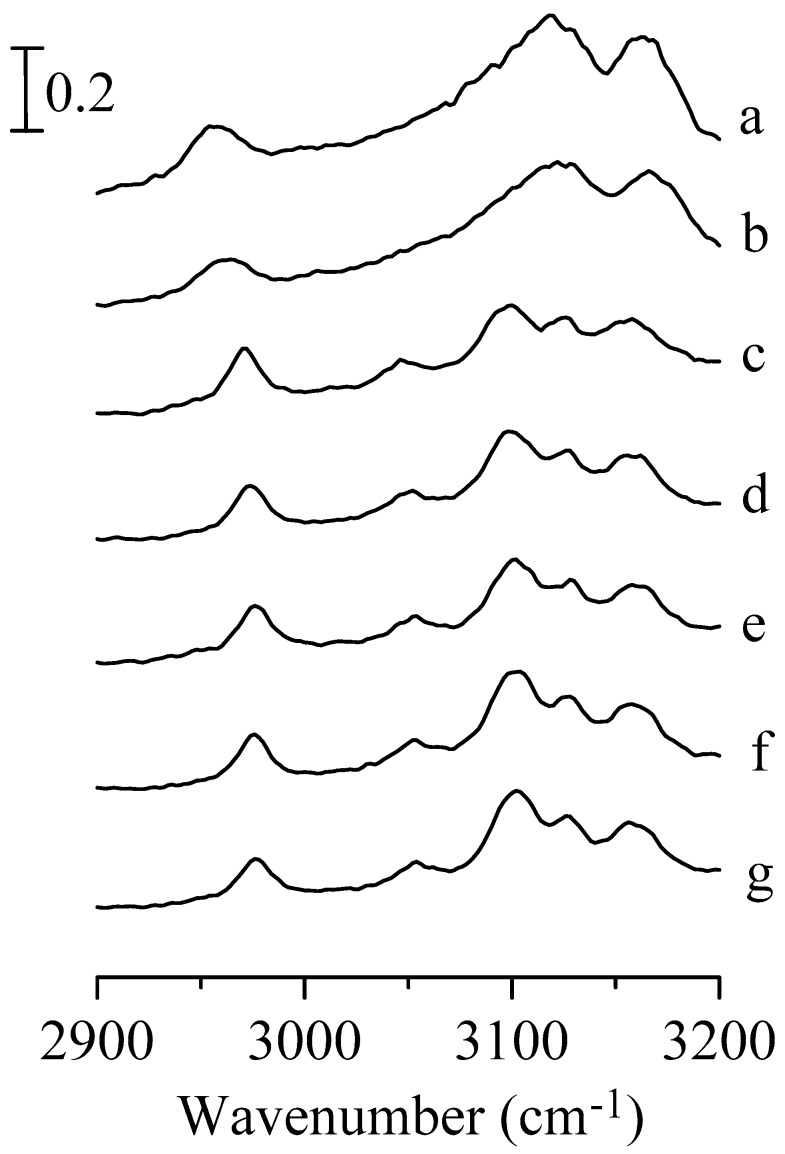
Infrared spectra of pure 1,3-dimethylimidazolium methyl sulfate obtained under ambient pressure (curve a) and at 0.4 (curve b), 0.7 (curve c), 1.1 (curve d), 1.5 (curve e), 1.8 (curve f), and 2.5 GPa (curve g).

Figure 1 shows the infrared spectra of pure 1,3-dimethylimidazolium methyl sulfate obtained under ambient pressure (curve a) and at 0.4 (curve b), 0.7 (curve c), 1.1 (curve d), 1.5 (curve e), 1.8 (curve f), and 2.5 GPa (curve g). As shown in Figure 1a, the spectrum reveals one alkyl C–H band at 2957 cm^−1^ and two imidazolium C–H bands at 3118 (C^2^–H stretching band) and 3162 cm^−1^ (C^4,5^–H stretching band). As the pressure was elevated to 0.4 GPa (Figure 1b), the alkyl C–H band shifted to 2963 cm^−1^ and the imidazolium C–H bands slightly shifted to 3121 and 3166 cm^−1^. In Figure 1b, the full width at half maximum (FWHM) of the C^2^–H stretching band and alkyl C–H band is about 28 cm^−1^ and 22 cm^−1^, respectively. Furthermore, there is a phase transition when the pressure is enhanced to 0.7 GPa. As shown in Figure 1c, the wavenumber of the alkyl C–H band increased to 2971 cm^−1^ with the FWHM decreasing to 15 cm^−1^. In addition, the C^2^–H stretching band was split into two absorption peaks at 3099 (with 25 cm^−1^ FWHM) and 3125 cm^−1^, the C^4,5^–H stretching band is red-shifted to 3157 cm^−1^. The frequency shifts observed in Figure 1c may be caused by the aggregation of ions when the pressure is increased. Moreover, the bandwidth narrowing of the vibrational absorptions can be attributed to the anisotropic environment in solid structures. Figure 1a–c reveals that the C^2^–H and C^4,5^–H bands tend to blue-shift in frequency (curve a and b) then red-shift (curve b and c) upon compression. It is instructive to note that the red-shift of the dominant C^2^–H band is about 22 cm^−1^ in Figure 1c in comparison to the red-shift of 8 cm^−1^ for the C^4,5^–H band. It can be observed from Figure 1c that the C–H hydrogen-bond interaction for imidazolium C^2^–H (with anions) is stronger than the imidazolium C^4,5^–H. The C^2^ of the imidazolium ring is located between two nitrogen atoms, which have large electronegativity. On the other hand, both C^4^–H and C^5^–H have weaker acidity as just one nitrogen atom is nearby, compared with C^2^–H. We do not observe a red-shift in the frequency for the alkyl C–H band in Figure 1c, indicating weak alkyl C–H hydrogen bonding compared to imidazolium C–H. Previous study has shown that the vibrational shifts in frequency are caused by factors such as electrostatic interactions, polarization, and steric repulsion between proton donors and acceptors for both red-shifting and blue-shifting hydrogen bonds [44,45]. Similar to our discontinuous frequency shift for the C^2^–H and C^4,5^–H vibrations, some studies have indicated that a strong electric field environment not only strengthens the hydrogen bonds between neutral molecules (such as methane) and water but also affects the lengths of the covalent bonds in the neutral molecules (shorter in low electric field and lengthening as the field increases) [44,45]. In addition, the infrared absorption bands show a mild blue-shift when the applied pressure was increased to over 0.7 GPa.

To obtain the amounts of ionic liquids in the mixtures [46,47], thermogravimetric analysis (TGA) measurements were performed (see Figure S2 in the Supplementary Materials, for example). The TGA temperature ranges from 30 °C to 600 °C, and the total weight loss at 500 °C represents the amount of ionic liquids in the mixtures. Based on our TGA results, the mixtures contain 41, 58, 43, and 66 wt % of ionic liquids for Al_2_O_3_/1,3-dimethylimidazolium methyl sulfate, mica/1,3-dimethylimidazolium methyl sulfate, Al_2_O_3_/1-ethyl-3-methylimidazolium trifluoromethanesulfonate, and mica/1-ethyl-3-methylimidazolium trifluoromethanesulfonate, respectively.

**Figure 2:**
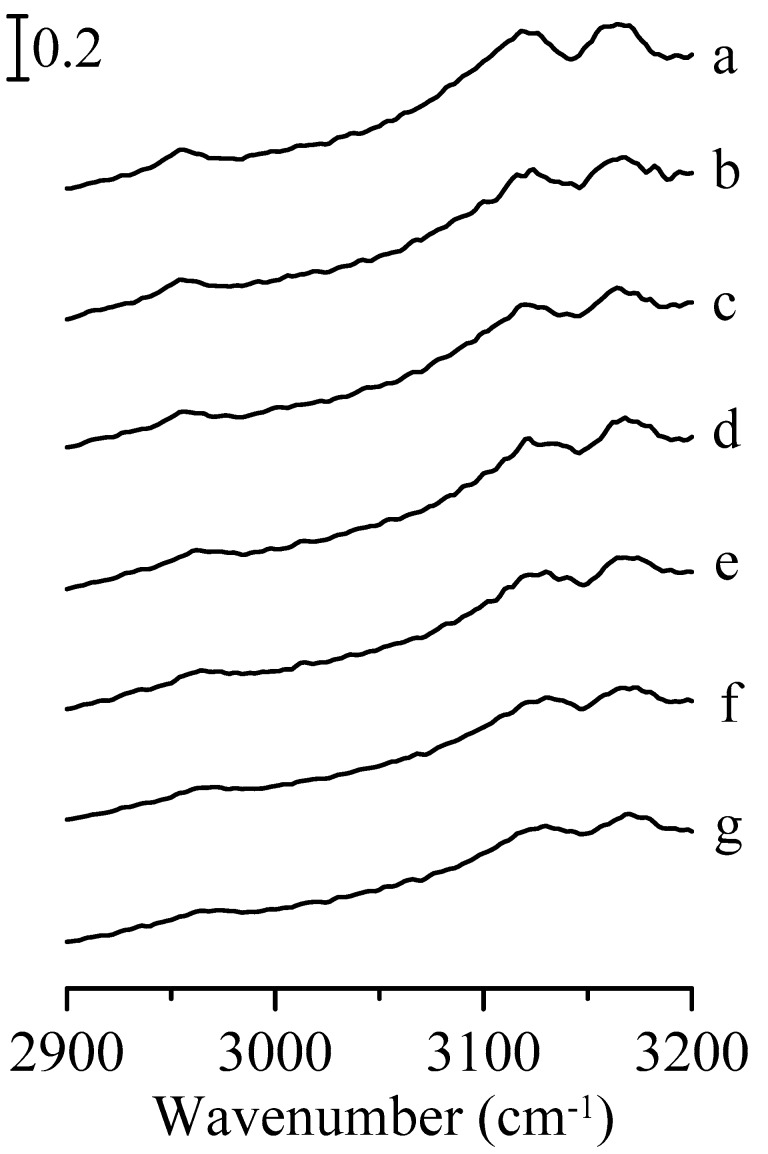
Infrared spectra of the Al_2_O_3_/1,3-dimethylimidazolium methyl sulfate mixture obtained under ambient pressure (curve a) and at 0.4 (curve b), 0.7 (curve c), 1.1 (curve d), 1.5 (curve e), 1.8 (curve f), and 2.5 GPa (curve g).

Figure 2 presents the infrared absorption spectra of an Al_2_O_3_/1,3-dimethylimidazolium methyl sulfate mixture under various pressures. It should be noted that the spectral features in Figure 2a are similar to those observed for the pure 1,3-dimethylimidazolium methyl sulfate in Figure 1a. As the pressure was increased to 0.7 GPa in Figure 2c, the C^2^–H and C^4,5^–H stretching bands of imidazolium are shifted to about 3122 and 3166 cm^−1^, respectively. The FWHM of the C^2^–H stretching bands are almost the same as the applied pressure is raised from ambient to 0.7 GPa. Splitting of the C^2^–H stretching band at 0.7 GPa is not observed in Figure 2c, unlike that seen in Figure 1c. The results in Figure 2 indicate that the presence of mesoporous aluminum oxide may perturb the local structure of the ionic liquid in the mixture under high pressure. The spectra in Figure 2d–g (pressure > 0.7 GPa) reveal no remarkable changes in spectral features except for further shifting in frequency.

Figure S1 (in the Supplementary Materials) shows the infrared spectra of a mica/1,3-dimethylimidazolium methyl sulfate mixture obtained under various pressures. There is only a single C^2^–H stretching band in Figure S1, similar to the one in Figure 2. The widths of the imidazolium C–H bands in Figure S1 do not show a significant sharpening trend. These features are almost identical to those in Figure 2. Based on the results in Figure 2 and Figure S1, both Al_2_O_3_ and mica can disturb the supramolecular assemblies of the cation–anion in the mixtures via pressure-enhanced interfacial interactions under high pressures.

**Figure 3:**
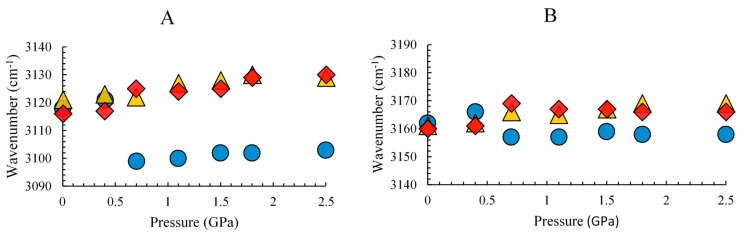
Pressure dependence of the imidazolium C–H stretching frequencies at (**A**) 3120 and (**B**) 3160 cm^−1^ of pure 1,3-dimethylimidazolium methyl sulfate (circles), Al_2_O_3_/1,3-dimethylimidazolium methyl sulfate mixture (triangles), and mica/1,3-dimethylimidazolium methyl sulfate mixture (diamonds).

The pressure dependence of the C–H stretching frequencies of the pure ionic liquid, mica/ionic liquid, and Al_2_O_3_/ionic liquid mixtures is plotted in Figure 3 to illustrate the frequency shift. In Figure 3A, the C^2^–H absorption for the pure ionic liquid is significantly red-shifted at 0.7 GPa due to the pressure-enhanced cooperative effect toward the “crystal-like” structure of the clusters. Previous studies have shown that hydrogen bond cooperativity, which is induced by concerted charge transfer, can cause the interaction of hydrogen bonds to be remarkably strengthened [48,49]. However, such a red-shift in frequency of the C^2^–H band is not observed for the mixtures with Al_2_O_3_ and mica (Figure 3A). Al_2_O_3_ and mica particles in the mixture seem to disturb the structure of the ionic liquid as the C^2^–H stretching bands for the mixture blue-shift and as the pressure is increased (Figure 3A). The Pressure-enhanced interfacial interactions between the cation and the solid surface may be one of reasons to explain these results. Similarly, a red-shift can also be observed for the C^4,5^–H stretching bands of pure ionic liquid at 0.7 GPa as illustrated by Figure 3B. However, the red-shifts in frequency are more significant for C^2^–H than for C^4,5^–H due to the acidity and stronger hydrogen bonding of C^2^–H. Hence, the C^2^–H stretching band is more sensitive to pressure variation compared to the C^4,5^–H stretching band.

**Figure 4:**
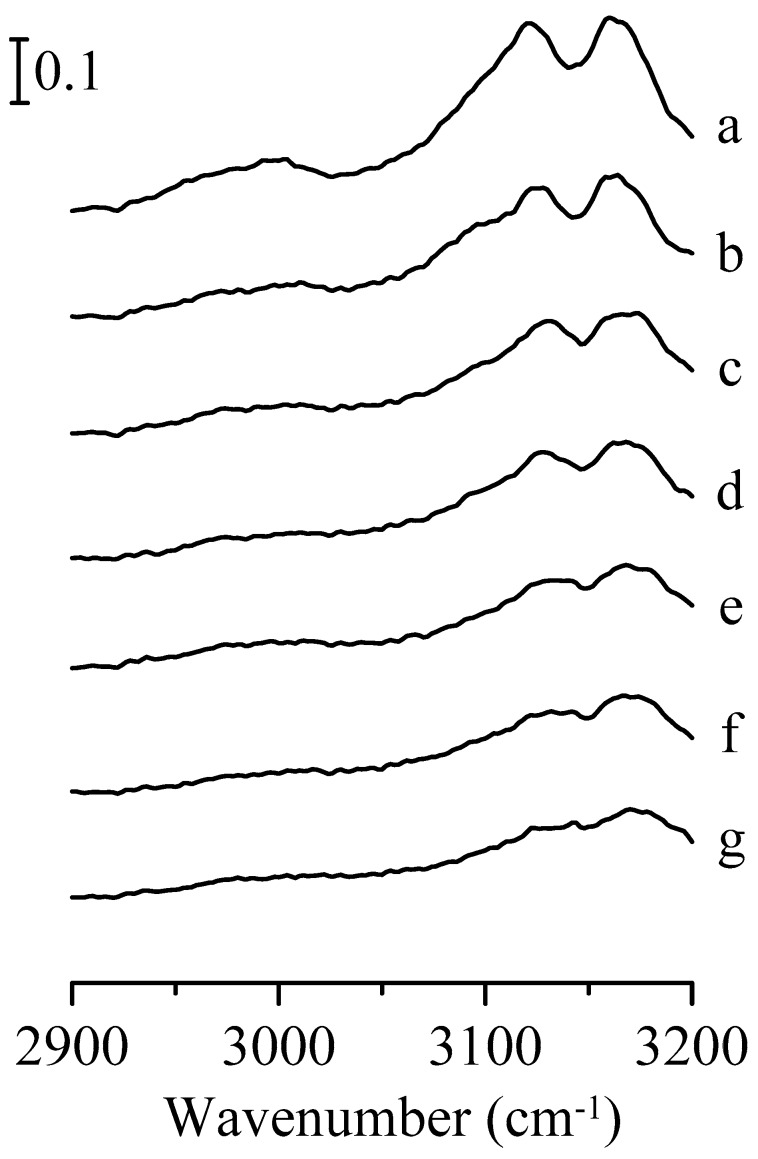
Infrared spectra of pure 1-ethyl-3-methylimidazolium trifluoromethanesulfonate obtained under ambient pressure (curve a) and at 0.4 (curve b), 0.7 (curve c), 1.1 (curve d), 1.5 (curve e), 1.8 (curve f), and 2.5 GPa (curve g).

Figure 4 illustrates the infrared spectra of pure 1-ethyl-3-methylimidazolium trifluoromethanesulfonate under different pressures. In Figure 4a, the side chain alkyl C–H vibrational band is at ~2985 cm^−1^ and the imidazolium C–H bands are at ~3121 and 3162 cm^−1^ (bandwidths are about 35 and 32 cm^−1^, respectively) associated with a minor shoulder at ~3100 cm^−1^ under ambient pressure. Previous study has revealed that the imidazolium C^2^–H bands can have two absorptions, that is, 3100 and 3121 cm^−1^. The existence of two C^2^–H absorptions indicates that there are two forms of imidazolium C–H local structure caused by the different sizes of the ion clusters in the ionic liquid. The absorption peaks at 3100 cm^−1^ and 3121 cm^−1^ may arise from the small clusters (isolated structures) and large clusters (associated structures), respectively [50]. At 0.4 GPa, the major C^2^–H stretching band (associated form) is blue-shifted to ~3125 cm^−1^ with around 17 cm^−1^ FWHM as shown in Figure 4b. As the pressure was elevated from 0.4 GPa to 2.5 GPa (from Figure 4b to Figure 4g), there were no drastic changes in the absorption peaks other than mild signal shifting and band broadening. In addition, the imidazolium C^2^–H band in Figure 4c does not have a splitting pattern, unlike that shown in Figure 1c.

**Figure 5:**
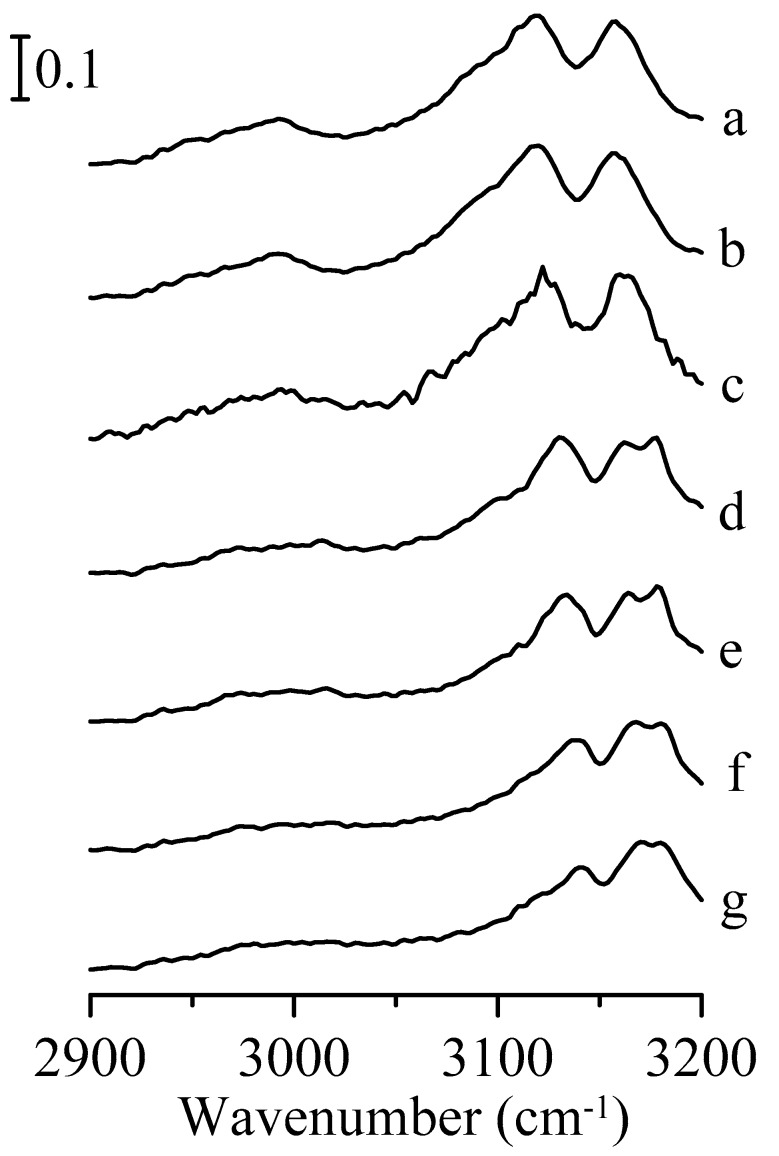
Infrared spectra of Al_2_O_3_/1-ethyl-3-methylimidazolium trifluoromethanesulfonate mixture obtained under ambient pressure (curve a) and at 0.4 (curve b), 0.7 (curve c), 1.1 (curve d), 1.5 (curve e), 1.8 (curve f), and 2.5 GPa (curve g).

Figure 5 reveals the infrared spectra of the Al_2_O_3_/1-ethyl-3-methylimidazolium trifluoromethanesulfonate mixture acquired under different pressures. At ambient pressure (Figure 5a), there is an unresolved broad alkyl C–H stretching band at ~2991 cm^−1^ and two imidazolium C–H stretching peaks at ~3119 and 3158 cm^−1^ associated with a minor shoulder at ~3097 cm^−1^, similar to the shoulder that appears in Figure 4a. The signal intensities of the imidazolium C–H bands are almost the same. As the pressure is increased to 1.1 GPa, as demonstrated in Figure 5d, the C^4,5^–H stretching band is split into two peaks at ~3163 and 3177 cm^−1^. The imidazolium C–H bands contain two doublets (two bands for C^2^–H, and the other two bands for C^4,5^–H). The imidazolium C^2^–H and C^4,5^–H absorptions of the associated structure are located at ~3121 and 3177 cm^−1^, respectively, whereas the imidazolium C–H absorptions of the isolated form appear at ~3100 and 3163 cm^−1^. The increase in intensity of the 3177 cm^−1^ band in Figure 5d suggests the partial switch from the isolated form to associated structures at high pressures. In addition, the major band for C^2^–H moved to ~3131 cm^−1^ and its bandwidth decreased from about 50 cm^−1^ to 29 cm^−1^ as the pressure increased from ambient to 1.1 GPa due to the decrease in the molar ratio of isolated/associated forms. The spectral changes in Figure 5d indicate that a phase transition may occur under the pressure of 1.1 GPa. In addition, this type of phase transition in Figure 5 is different from that for pure 1-ethyl-3-methylimidazolium trifluoromethanesulfonate at high pressures (Figure 4). The presence of mesoporous aluminum oxide seems to force the ionic liquid to array in another way, causing the C^2^–H stretching absorption attributed to isolated structures to decrease in intensity. The spectroscopic features in Figure 5d–g are considerably similar except for a slight decrease in intensity for the 3179 cm^−1^ band (associated C^4,5^–H structure).

**Figure 6:**
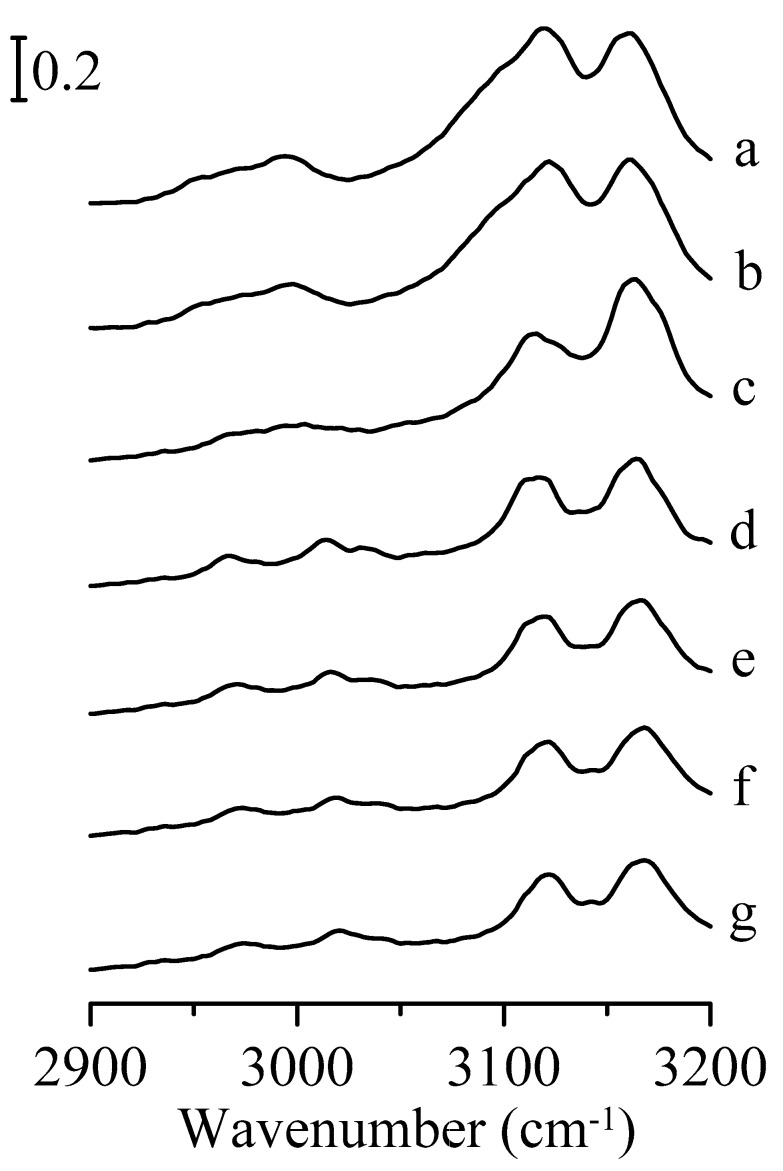
Infrared spectra of mica/1-ethyl-3-methylimidazolium trifluoromethanesulfonate mixture obtained under ambient pressure (curve a) and at 0.4 (curve b), 0.7 (curve c), 1.1 (curve d), 1.5 (curve e), 1.8 (curve f), and 2.5 GPa (curve g).

Figure 6 illustrates the infrared spectra of the mica/1-ethyl-3-methylimidazolium trifluoromethanesulfonate mixture obtained under various pressures. As shown in Figure 6a, there is one alkyl C–H stretching band at ~2994 cm^−1^ and two major imidazolium C–H stretching peaks at ~3119 (C^2^–H in the associated form) and 3159 cm^−1^ (C^4,5^–H) accompanied with a shoulder at ~3100 cm^−1^ (C^2^–H in the isolated form) at normal pressure. At 0.4 GPa, the major imidazolium bands are shifted to ~3121 and 3162 cm^−1^ with approximately 21 and 23 cm^−1^ bandwidths, respectively (Figure 6b). Moreover, the C^2^–H band for the associated form dramatically decreases in intensity at 0.7 GPa as revealed in Figure 6c. At this pressure, the dominant C^2^–H stretching band is switched to ~3116 cm^−1^ (C^2^–H in isolated form) with the bandwidth decreasing to ~19 cm^−1^. As the pressure is increased to 1.1 GPa, as shown in Figure 6d, the absorption of the imidazolium bands reveals no significant shifts in frequency. Furthermore, the side chain alkyl C–H stretching band is split into three resolved peaks. These results imply the appearance of a phase transition, and the formation of alkyl C–H hydrogen bonds may provide compensatory stability (Figure 6d). Moreover, this type of phase transition is remarkably different from the one shown in Figure 5d. For the porous Al_2_O_3_/1-ethyl-3-methylimidazolium trifluoromethanesulfonate under high pressure in Figure 5d, the dominant C^2^–H stretching band arises from associated structures locating at ~3131 cm^−1^ at 1.1 GPa. However, this peak does not appear in Figure 6d for the mica mixture and the C^2^–H stretching band originating from isolated structures (~3115 cm^−1^) is the major peak in Figure 6d. In addition, the doublet of C^4,5^–H stretches in Figure 6d does not separate as clearly as the peaks shown in Figure 5d, indicating a reduction in the intensity of the associated form in Figure 6d. These tremendous variations reflect that the surface properties of materials (Al_2_O_3_, mica) added into the mixtures can affect the interfacial structures and hydrogen-bonding patterns of the ionic liquid in different ways under high pressures.

**Figure 7:**
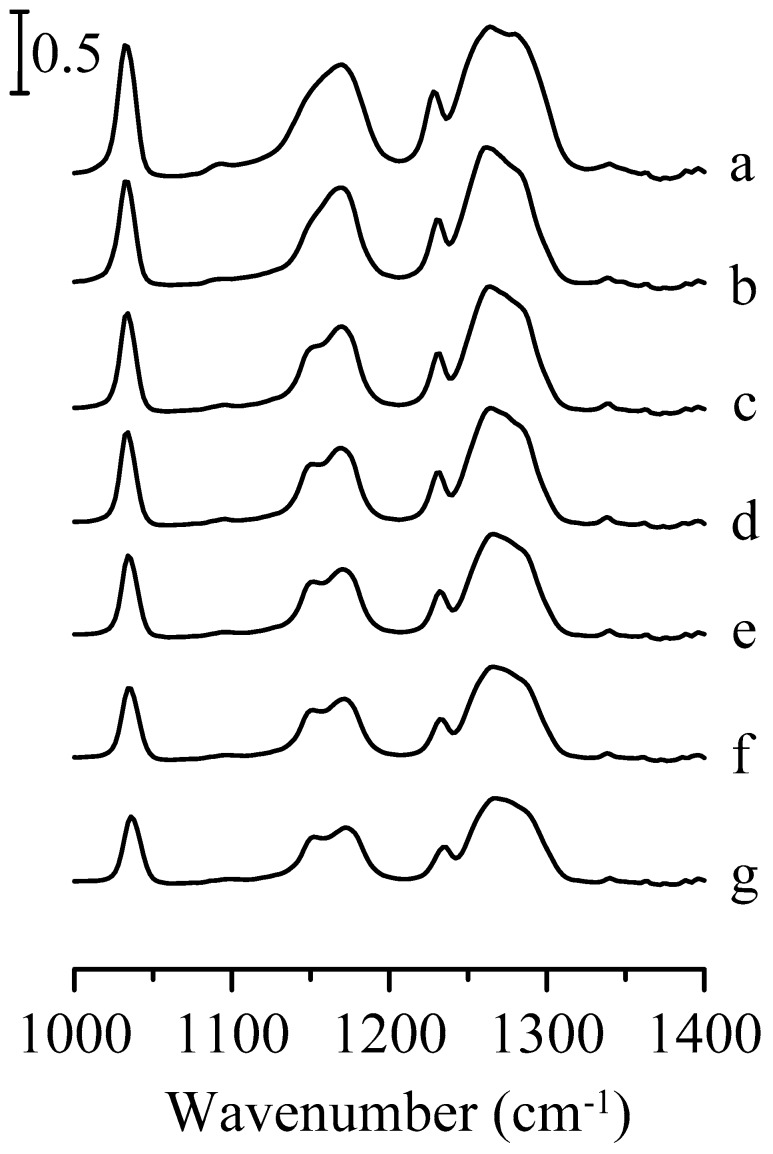
Infrared spectra of pure 1-ethyl-3-methylimidazolium trifluoromethanesulfonate obtained under ambient pressure (curve a) and at 0.4 (curve b), 0.7 (curve c), 1.1 (curve d), 1.5 (curve e), 1.8 (curve f), and 2.5 GPa (curve g).

Figure 7 displays the infrared spectra of pure 1-ethyl-3-methylimidazolium trifluoromethanesulfonate in the spectral range between 1000 and 1400 cm^−1^. In this region, the strong absorption bands are mostly attributed to the vibrational modes of trifluoromethanesulfonate. At ambient pressure (Figure 7a), the symmetric and asymmetric stretching bands of the SO_3_ group are at ~1033 and 1271 cm^−1^, respectively [22,35]. The peaks located at about 1164 and 1229 cm^−1^ in Figure 7a are assigned to CF_3_ asymmetric and symmetric stretching vibrations, respectively [35]. As the pressure is raised to 0.7 GPa (Figure 7c), the CF_3_ asymmetric stretching band is resolved into two peaks at ~1152 and 1172 cm^−1^. On the other hand, the splitting of the SO_3_ asymmetric band is not significant at this pressure.

**Figure 8:**
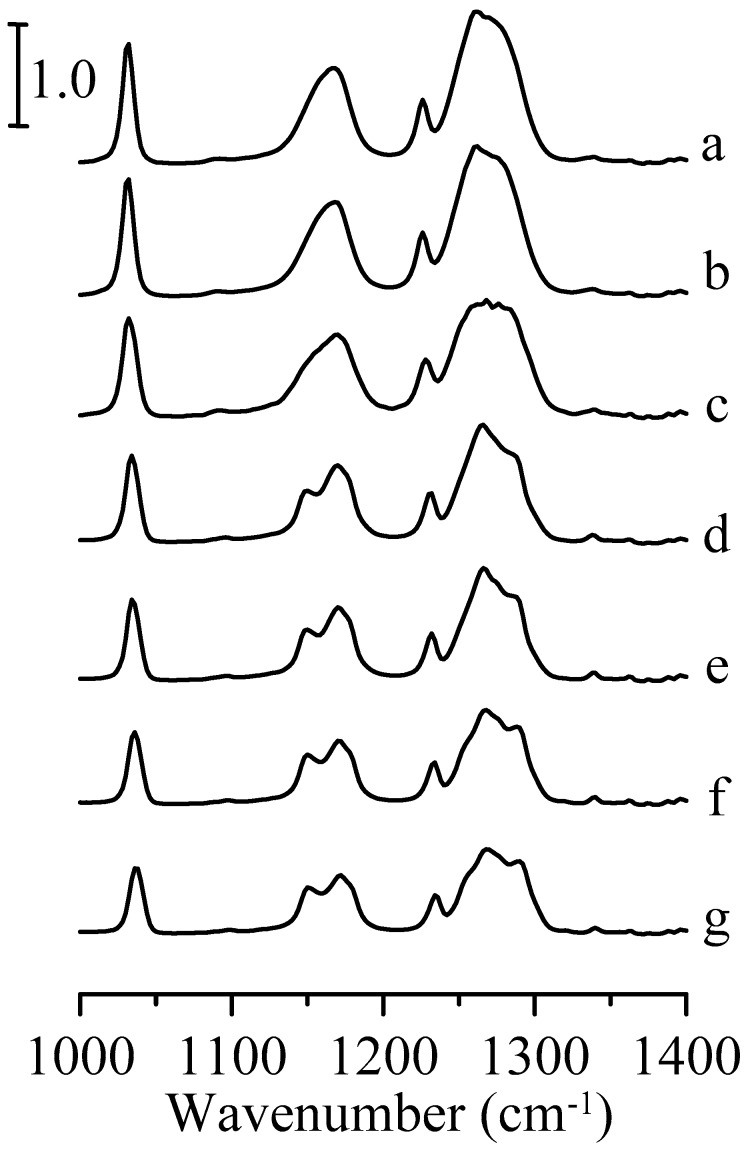
Infrared spectra of Al_2_O_3_/1-ethyl-3-methylimidazolium trifluoromethanesulfonate mixture obtained under ambient pressure (curve a) and at 0.4 (curve b), 0.7 (curve c), 1.1 (curve d), 1.5 (curve e), 1.8 (curve f), and 2.5 GPa (curve g).

Figure 8 shows the infrared spectra of the Al_2_O_3_/1-ethyl-3-methylimidazolium trifluoromethanesulfonate mixture acquired under different pressures. The CF_3_ asymmetric stretching band is split into two peaks at 1.1 GPa (Figure 8d). The presence of Al_2_O_3_ particles seems to perturb the structure of the ionic liquid in the mixture since the splitting occurs at a higher pressure (1.1 GPa) for the mixture compared to the pressure of 0.7 GPa for pure 1-ethyl-3-methylimidazolium trifluoromethanesulfonate. Based on the results in Figure 8, the interfacial interactions between ionic liquid and porous Al_2_O_3_ may play a non-negligible role.

**Figure 9:**
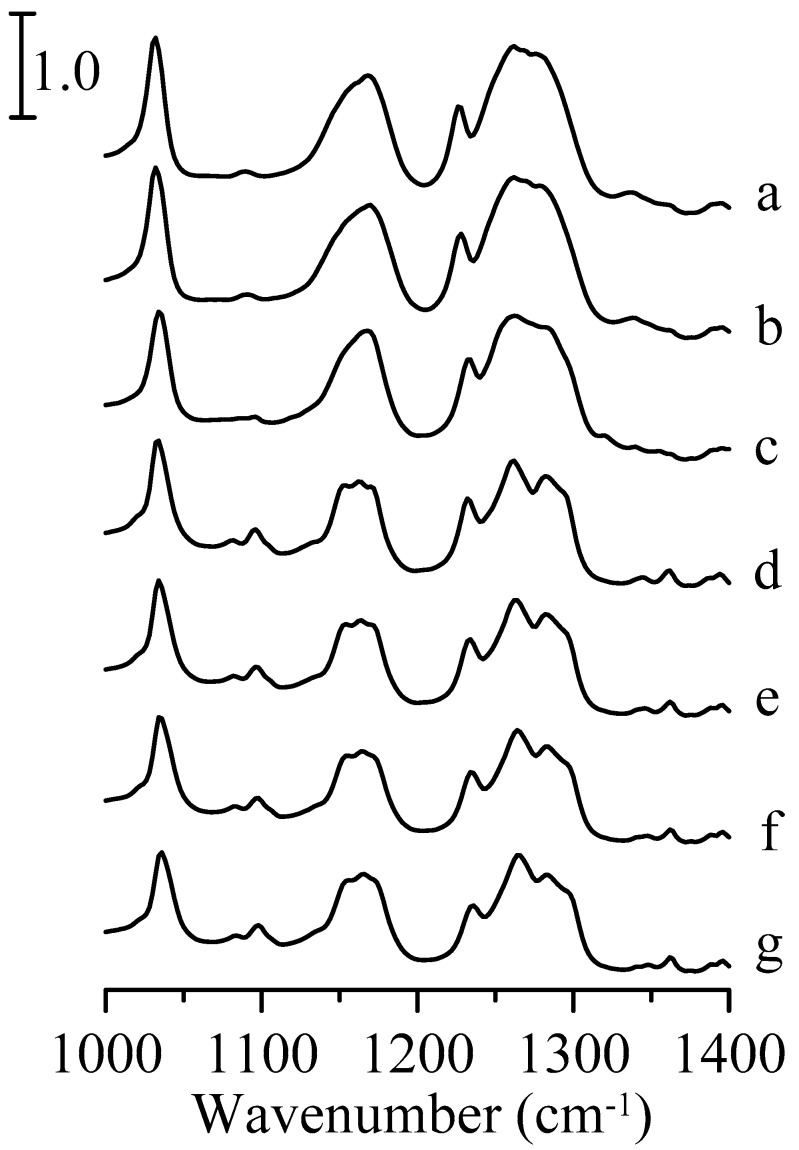
Infrared spectra of mica/1-ethyl-3-methylimidazolium trifluoromethanesulfonate mixture obtained under ambient pressure (curve a) and at 0.4 (curve b), 0.7 (curve c), 1.1 (curve d), 1.5 (curve e), 1.8 (curve f), and 2.5 GPa (curve g).

Figure 9 illustrates the infrared spectra of the mica/1-ethyl-3-methylimidazolium trifluoromethanesulfonate mixture obtained under various pressures. Both the SO_3_ and CF_3_ asymmetric stretching bands are split at 1.1 GPa (Figure 9d). The splitting for the SO_3_ asymmetric absorption is clearer than the splitting for CF_3_. It should be noted that the spectral features of splitting in Figure 9d are different from those in Figure 8d. Compression leads to dramatic spectral changes in Figure 9d–g, as a new band at ca. 1100 cm^−1^ arose. We note that no more vibration modes exist in this region (see Figure 7d–g and Figure 8d–g). This new spectral feature located at 1100 cm^−1^ in Figure 9d–g should be assumed to arise from the structural reorganization of SO_3_ groups induced by the interactions between ionic clusters and mica surfaces, but the details remain unclear. Previous sum-frequency generation spectroscopic study indicates that two strong SO_3_ peaks at 1047 and 1101 cm^−1^ as well as one weak shoulder at 1037 cm^−1^ were observed at 1400 mV, and the strong SO_3_ peaks at 1101 cm^−1^ was assigned to anion (trifluoromethanesulfonate) strongly absorbed on surfaces [22]. The results in Figure 8 and Figure 9 indicate that the subtle difference in the disturbance caused by interfacial interactions with surfaces of porous Al_2_O_3_ and mica can be probed by our high-pressure method.

We notice that a debate exists about the interpretation of IR spectra in the frequency region of imidazolium C-H stretching vibrations [50,51,52,53]. The complicated spectral feature of the absorption band at ca. 3119 cm^−1^ results from the C^2^–H stretching vibrations and Fermic resonances of the C-H stretching vibrations with overtones [53]. Previous studies focus either on Fermic-resonance interaction [51] of isolated imidazolium cation or hydrogen-bonding in a cluster model [50]. This study indicates the non-negligible role of the cluster model in ionic liquid/solid systems. The vibrational frequency of absorption bands in Figures are listed in Table S1 (in the Supplementary Materials).

## 4. Conclusions

A pressure-enhanced cooperative effect has been observed for pure 1,3-dimethylimidazolium methyl sulfate under high pressures. Solid particles (Al_2_O_3_ and mica) may reduce the cooperative effect caused by the local structure change of 1,3-dimethylimidazolium methyl sulfate under high pressures due to pressure-enhanced interfacial interactions. The infrared spectra of pure 1-ethyl-3-methylimidazolium trifluoromethanesulfonate and 1-ethyl-3-methylimidazolium trifluoromethanesulfonate/solid particle (Al_2_O_3_ and mica) mixtures show that the local structures of ionic liquids can be affected by the specific properties of the solid surfaces under high pressures. In the Al_2_O_3_/1-ethyl-3-methylimidazolium trifluoromethanesulfonate mixture, the ionic liquid tends to array in associated form at pressures above 1.1 GPa. For the mica/1-ethyl-3-methylimidazolium trifluoromethanesulfonate mixture, the preferred structure takes an isolated form under high pressures. These results demonstrate that high-pressure infrared spectroscopy is an effective tool for probing interfacial interactions in mixtures.

## Supplementary Materials

The following are available online at https://www.mdpi.com/2079-4991/9/3/373/s1, Figure S1: Infrared spectra of the mica/1,3-dimethylimidazolium methyl sulfate mixture obtained under ambient pressure (curve a) and at 0.4 (curve b), 0.7 (curve c), 1.1 (curve d), 1.5 (curve e), 1.8 (curve f), and 2.5 GPa (curve g), Figure S2: TGA measurement of mica/1-ethyl-3-methylimidazolium trifluoromethanesulfonate mixture, Table S1: The peak top positions of vibrational peaks.
