# Supplementary material for: Utilizing Infrared Spectroscopy to Analyze the Interfacial Structures of Ionic Liquids/Al2O3 and Ionic Liquids/Mica Mixtures under High Pressures
Source: Nanomaterials (Basel). 2019 Mar 5;9(3):373. doi: 10.3390/nano9030373 (PMC6473959; doi:10.3390/nano9030373)
Supplement: Supplementary file 1 [file nanomaterials-09-00373-s001.pdf]

Supplementary Materials

Utilizing Infrared Spectroscopy to Analyze the Interfacial Structures of Ionic Liquids/Al_2O_3 and Ionic Liquids/Mica Mixtures under High Pressures

Yen-Hsu Chang [1], Hai-Chou Chang [1,*] and Yen-Pei Fu [2]

[1] Department of Chemistry, National Dong Hwa University, Shoufeng, Hualien 974, Taiwan; 410412017@gms.ndhu.edu.tw

[2] Department of Materials Science and Engineering, National Dong Hwa University, Shoufeng, Hualien 974, Taiwan; ypfu@gms.ndhu.edu.tw

* Correspondence: hcchang@gms.ndhu.edu.tw; Tel.: +886-3-8903585

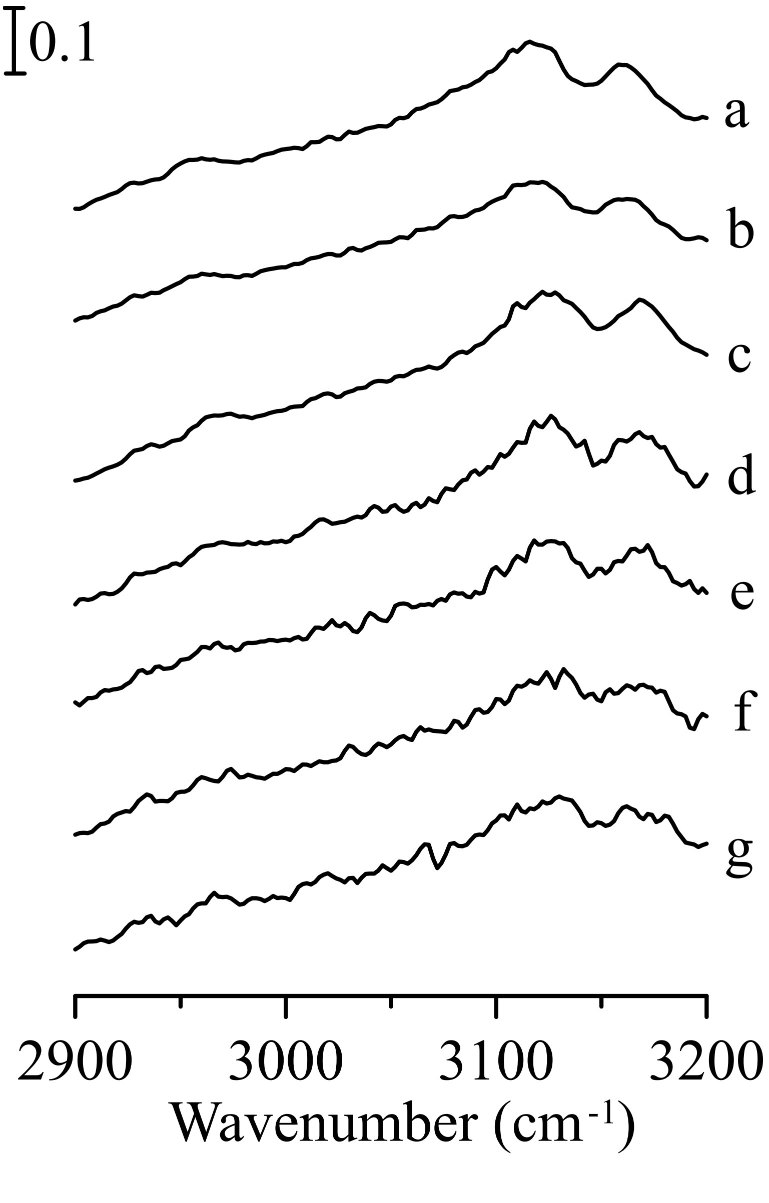

Figure S1. Infrared spectra of mica/1,3-dimethylimidazolium methyl sulfate mixture obtained under ambient pressure (curve a) and at 0.4 (curve b), 0.7 (curve c), 1.1 (curve d), 1.5 (curve e), 1.8 (curve f), and 2.5 GPa (curve g).

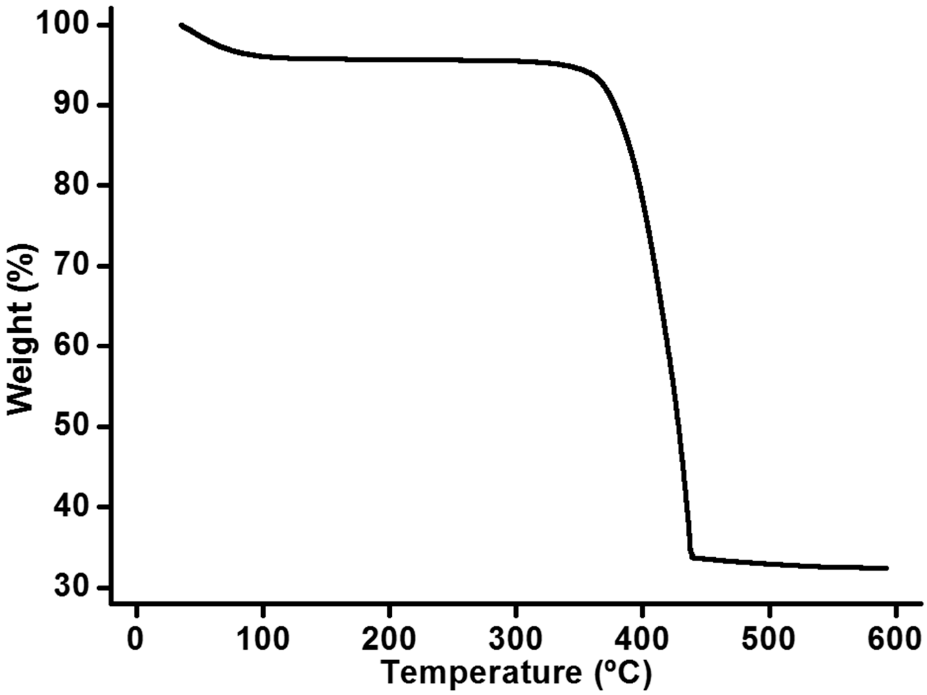

Figure S2. TGA measurement of mica/1-ethyl-3-methylimidazolium trifluoromethanesulfonate mixture

Table S1. The peak top positions of vibrational peaks

Figure.1	Wavenumber (cm^{-1})					
a	2957	3118		3162		
b	2963	3121		3166		
c	2971	3099	3125	3157		
d	2974	3100	3127	3157		
e	2977	3102	3128	3159		
f	2976	3102	3126	3158		
g	2977	3103	3126	3158		
Figure.2	Wavenumber (cm^{-1})					
a	2956	3121	3161			
b	2958	3123	3162			
c	2967	3122	3166			
d	2967	3126	3166			
e	2968	3128	3167			
f	2971	3130	3169			
g	2978	3129	3169			
Figure.4	Wavenumber (cm^{-1})					
a	3121	3163				
b	3125	3163				
c	3130	3167				
d	3130	3167				
e	3134	3169				
f	3135	3169				
g	3141	3171				
Figure.5	Wavenumber (cm^{-1})					
a	2991	3119	3158			
b	2991	3118	3157			
c		3122	3162			
d		3131	3163	3177		
e		3133	3164	3179		
f		3138	3167	3179		
g		3141	3171	3179		

Figure.6	Wavenumber (cm^{-1})					
a	2994	3119	3159			
b	2996	3121	3162			
c		3116	3164			
d		3116	3163			
e		3118	3165			
f		3120	3167			
g		3121	3167			
Figure.7	Wavenumber (cm^{-1})					
a	1033	1164		1229	1271	
b	1033	1165		1231	1269	
c	1034	1152	1172	1232	1270	
d	1034	1151	1171	1232	1270	
e	1035	1151	1172	1233	1271	
f	1035	1151	1172	1233	1272	
g	1037	1153	1174	1235	1273	
Figure.8	Wavenumber (cm^{-1})					
a	1032	1167		1226	1268	
b	1031	1168		1226	1268	
c	1033	1169		1228	1270	
d	1034	1151	1170	1231	1266	1286
e	1035	1151	1170	1232	1267	1286
f	1036	1151	1171	1233	1269	1287
g	1037	1151	1172	1235	1269	1288
Figure.9	Wavenumber (cm^{-1})					
a	1032	1164	1227	1269		
b	1032	1164	1228	1269		
c	1034	1164	1233	1270		
d	1034	1161	1233	1262	1283	
e	1035	1162	1234	1264	1283	
f	1036	1162	1235	1265	1283	
g	1036	1163	1236	1266	1283	
